# The Tenerife moment in health professions’ education: a conceptual call to navigate uncertainty

**DOI:** 10.3389/fmed.2026.1752213

**Published:** 2026-04-10

**Authors:** Jose Knopfholz, Patricia Tempski, Leandro Zen Karam, Lucas Bertelli Fogaça, Milton Arruda Martins

**Affiliations:** 1 Escola de Medicina e Ciências da Vida da Pontifícia Universidade Católica do Paraná, Curitiba, Brazil; 2 Faculdade de Medicina da Universidade de São Paulo, São Paulo, Brazil; 3 Escola Politécnica da da Pontifícia Universidade Católica do Rio Grande do Sul, Porto Alegre, Brazil

**Keywords:** aviation, communication, Crew Resource Management, medical education, uncertainty

## Abstract

The 1977 Tenerife airport disaster, the deadliest accident in aviation history, was not caused by mechanical failure but by ambiguity, hierarchy, and miscommunication under conditions of uncertainty. Aviation’s response to this tragedy marked a systemic turning point: the development of Crew Resource Management (CRM), standardized communication protocols, simulation-based human factors training, and regulatory reforms that embedded uncertainty management into professional preparation. This article introduces the concept of a “Tenerife moment” in health professions education—a critical juncture at which systems must recognize and respond to latent vulnerabilities before catastrophe compels change. We argue that uncertainty is not a deficit in healthcare practice but an inherent and defining condition. Yet medical and health professions curricula often privilege technical certainty, reward singular correct answers, and perpetuate hidden curricula that equate confidence with competence. As healthcare environments grow increasingly complex—shaped by rapid technological innovation, evolving evidence, global crises, and interprofessional dynamics—graduates are frequently underprepared to communicate, decide, and lead under ambiguity. Drawing conceptual lessons from aviation’s transformation, we explore how health professions education might more explicitly address uncertainty through CRM-informed teamwork training, standardized communication tools, simulation of ambiguous scenarios, interprofessional learning, faculty development that models vulnerability, and cultural alignment supported by institutional regulation. We also acknowledge potential tensions, including learner anxiety and faculty discomfort, underscoring the need for thoughtful implementation. Recognizing uncertainty as a core professional competency may represent healthcare’s own Tenerife moment—an opportunity to foster cultures of voice, shared responsibility, and adaptive expertise.

## Introduction: when fog becomes fatal

On the afternoon of March 27, 1977, Los Rodeos Airport in Tenerife was shrouded in fog so thick that the control tower could barely see the runway. The island had become unexpectedly congested after a terrorist incident diverted multiple flights from nearby Gran Canaria. Two of the largest aircraft in existence at the time—KLM Flight 4,805 and Pan Am Flight 1736, both Boeing 747 s—were forced to land on a small regional airfield never designed to accommodate such giants.

Inside the KLM cockpit sat one of the airline’s most senior pilots and a revered instructor. His reputation was legendary: he was the face of KLM’s training program, featured in advertisements, and considered almost untouchable in stature. Yet that day, he faced growing pressure. Hours of delays had stretched crew duty limits, passengers were restless, and worsening weather threatened further disruption. The Pan Am crew, equally fatigued, waited on the same runway.

The fog thickened. Visibility dropped to less than 300 meters. Air traffic controllers were overwhelmed, communications were imprecise, and radar could not reveal what was unfolding on the tarmac. Then came the fatal sequence: convinced he had clearance, the KLM captain pushed the throttles forward. His first officer hesitated—“Wait, are we cleared?”—but the captain’s authority silenced him. The flight engineer attempted to challenge—“Is he not clear?”—but his words were brushed aside. Seconds later, the KLM aircraft accelerated into the fog, colliding with the Pan Am aircraft still taxiing on the runway. Within minutes, 583 lives were lost.

The Tenerife disaster remains the deadliest accident in aviation history. What is most striking is not the magnitude of loss, but the nature of its causes. There was no mechanical failure, no act of terrorism, and no single catastrophic error. Instead, the tragedy emerged from uncertainty: uncertainty in communication (“We are at takeoff” interpreted as clearance), uncertainty in visibility, and uncertainty shaped by hierarchy, where junior crew members doubted but did not speak forcefully enough. A fog of ambiguity—both literal and metaphorical—proved more deadly than any technical malfunction (1).

Aviation could have chosen denial, attributing Tenerife to an unfortunate convergence of weather and human error. Instead, it chose transformation. Investigators concluded that the accident represented a systemic failure—of communication protocols, of crew dynamics, and of training (1). This recognition became aviation’s turning point, its Tenerife moment. Out of the tragedy emerged Crew Resource Management (CRM), standardized phraseology, simulation-based training for crisis management, and, above all, a culture that legitimized speaking up (2, 3). Today, more than four decades later, aviation is one of the safest industries in the world precisely because it learned to face uncertainty rather than conceal it (2).

The comparison between aviation and medicine is not new. For decades, scholars have pointed to CRM as a model for teamwork and simulation as a tool for patient safety (3–5). What is often overlooked, however, is the central role of uncertainty in this transformation. Tenerife was, at its core, a crisis of ambiguity: unclear communication, degraded situational awareness, and hierarchical hesitation. Aviation responded not by attempting to eliminate uncertainty, but by embedding it into training—teaching professionals how to act responsibly within it (1, 3).

Medical education has not yet made this leap. Curricula are saturated with technical content but often treat uncertainty as an undesirable weakness rather than a defining professional condition (6, 7). Assessment systems reinforce the illusion: examinations reward singular certainty, rarely encouraging learners to articulate probabilities, acknowledge limits of knowledge, or negotiate ambiguity with patients and colleagues (6, 8, 9). The hidden curriculum whispers that confidence matters more than caution (10).

Meanwhile, the reality of healthcare is becoming increasingly uncertain. Rapid technological advances, shifting guidelines, complex patient populations, and global crises—from pandemics to climate-related emergencies—demand professionals capable of thinking and acting under ambiguity (6, 7). Without explicit preparation, graduates are left to navigate uncertainty alone, often internalizing stress, anxiety, and burnout (7).

We are, in other words, standing in our own fog. The parallels with Tenerife are uncomfortable: pressure, ambiguity, hierarchy, and silence (2, 11). The question is whether health professions education will wait for its own catastrophic “collision” before acting—or whether it will recognize this moment as a call for systemic change.

## Conceptual clarifying note: what do we mean by “uncertainty”?

Throughout this manuscript, uncertainty is understood not as error or lack of competence, but as an inherent condition of healthcare practice. It refers to situations in which available information is incomplete, evolving, or ambiguous, and in which outcomes cannot be predicted with confidence. In clinical contexts, uncertainty may be diagnostic (unclear or conflicting data), prognostic (unpredictable disease trajectories), or therapeutic (multiple reasonable options with different risks, benefits, and value implications).

Importantly, uncertainty must be distinguished from related concepts. Risk refers to situations in which probabilities are known or estimable, whereas error implies deviation from accepted standards. Much of everyday clinical work unfolds precisely in spaces where probabilities are indeterminate and standards are context-dependent. Treating uncertainty as a weakness rather than a professional domain contributes to cultures of false certainty, silence, and hierarchical inhibition.

## Lessons from Tenerife and aviation’s transformation

The lessons drawn from Tenerife and aviation’s transformation are not presented here as prescriptive solutions to be directly transferred into healthcare. Rather, they serve as conceptual anchors for reflecting on how health professions education might more explicitly address uncertainty, hierarchy, and communication.

### From catastrophe to catalyst

The Tenerife disaster marked aviation’s darkest hour, but also its most transformative. Investigators concluded that the crash resulted not from a single mistake, but from systemic failures of communication, culture, and training (1, 2). Aviation resisted the temptation to scapegoat individuals and instead embraced systemic learning. This orientation became the foundation for its unprecedented safety record (2).

Healthcare, by contrast, often continues to frame adverse events as individual failures. Mortality and morbidity conferences may unintentionally reinforce blame rather than foster collective learning (11). For healthcare to experience its own Tenerife moment, it must similarly embrace systemic accountability.

### Crew Resource Management: making voice possible

Perhaps the most consequential outcome of Tenerife was the development of CRM. Aviation training expanded beyond technical skills to include leadership, communication, teamwork, situational awareness, and decision-making under uncertainty (3). CRM asserted a simple but revolutionary principle: safety is a shared responsibility, regardless of rank.

The parallels in healthcare are evident. A resident hesitant to challenge an attending mirrors the first officer at Tenerife who doubted clearance. A nurse reluctant to interrupt a surgeon echoes the flight engineer who questioned but retreated (2). Without deliberate training in communication and authority gradients, silence persists (10, 11).

This intersection between aviation and healthcare and the importance of CRM has been particularly visible in the field of human factors. A landmark example is the case of Elaine Bromiley, whose death during a routine surgical procedure exposed profound failures in communication, hierarchy, and situational awareness. Following this event, her husband, Martin Bromiley, a commercial airline pilot, applied principles drawn directly from aviation—including systems thinking, non-technical skills, and crew communication—to analyze the incident and advocate for systemic change in healthcare. The Bromiley case has since become a touchstone in patient safety literature, illustrating how aviation-derived human factors concepts can illuminate latent vulnerabilities in clinical systems.

### Standardized communication: reducing ambiguity

Aviation learned that communication under uncertainty must be explicit and standardized. After Tenerife, ambiguous radio exchanges were replaced by mandatory phraseology, dramatically reducing misinterpretation (1). In healthcare, communication remains comparatively informal. Tools such as SBAR^®^ (Situation, Background, Assessment, Recommendation) exist but are inconsistently applied (4).

Training must also address patient-facing uncertainty. Learners should practice explaining probabilities, acknowledging limits, and engaging patients in shared decision-making (8, 9). Evidence suggests that patients value honest uncertainty when delivered with empathy, whereas concealment erodes trust (8).

### Simulation: practicing uncertainty

Post-Tenerife, simulation became a laboratory for human factors training in aviation, exposing crews to high-pressure, ambiguous scenarios (5). Healthcare simulation, though widespread, often remains technically focused. Few curricula immerse learners in scenarios without a single correct answer—the reality of clinical practice (5–7).

### Regulation and cultural change

Aviation’s transformation was reinforced by regulation. CRM became mandatory, not optional (1, 2). In healthcare, uncertainty training is rarely enforced by accrediting bodies (6, 8). Without systemic reinforcement, educational reforms remain fragile (9).

### Interprofessional education

Uncertainty is rarely resolved by one profession alone. Interprofessional education allows learners to navigate ambiguity collectively through shared cases, simulations, and debriefings (10, 11), reducing isolation and fostering shared responsibility.

### Faculty development and modeling vulnerability

Faculty shape learners’ professional identities. When educators acknowledge uncertainty and invite dialogue, they model professionalism (7, 8). Faculty development must therefore legitimize vulnerability as a pedagogical asset.

### Addressing the hidden curriculum

Formal reforms will fail if the hidden curriculum remains unchanged. Learners notice when uncertainty is preached but punished (10, 12). Cultural change requires alignment of leadership, rewards, and role modeling (13, 14).

## Limitations, tensions, and unintended consequences

Emphasizing uncertainty in education is not without risk. Poorly facilitated discussions may heighten anxiety, undermine confidence, or foster decision paralysis. Encouraging speaking up without psychological safety may expose learners to social or professional harm. Faculty themselves may struggle to model uncertainty in cultures that reward decisiveness. These tensions highlight the need for careful curricular design, faculty preparation, and institutional alignment.

## Final reflections

The story of Tenerife is sobering not because it was unique, but because it was avoidable. Aviation chose to treat it as a turning point, acknowledging that uncertainty could not be eliminated but could be managed through culture, communication, training, and regulation (1–3).

Healthcare operates in environments defined by ambiguity and complexity, yet educational systems often continue to train as though certainty were the norm (6, 7). Health professions education may be approaching its own Tenerife moment—one that calls for explicit recognition of uncertainty as a core professional competency. Patients deserve professionals trained not only in the science of disease, but in the art of navigating uncertainty together.

## Data Availability

The original contributions presented in the study are included in the article/supplementary material, further inquiries can be directed to the corresponding author.
